# Natural language processing systems for extracting information from electronic health records about activities of daily living. A systematic review

**DOI:** 10.1093/jamiaopen/ooae044

**Published:** 2024-05-24

**Authors:** Yvonne Wieland-Jorna, Daan van Kooten, Robert A Verheij, Yvonne de Man, Anneke L Francke, Mariska G Oosterveld-Vlug

**Affiliations:** Netherlands Institute for Health Services Research (Nivel), Utrecht, Postbus 1568, 3500 BN, The Netherlands; Tranzo, School of Social Sciences and Behavioural Research, Tilburg University, Tilburg, Postbus 90153, 5000 LE, The Netherlands; Netherlands Institute for Health Services Research (Nivel), Utrecht, Postbus 1568, 3500 BN, The Netherlands; Netherlands Institute for Health Services Research (Nivel), Utrecht, Postbus 1568, 3500 BN, The Netherlands; Tranzo, School of Social Sciences and Behavioural Research, Tilburg University, Tilburg, Postbus 90153, 5000 LE, The Netherlands; Netherlands Institute for Health Services Research (Nivel), Utrecht, Postbus 1568, 3500 BN, The Netherlands; Netherlands Institute for Health Services Research (Nivel), Utrecht, Postbus 1568, 3500 BN, The Netherlands; Department of Public and Occupational Health, Location Vrije Universiteit Amsterdam, Amsterdam UMC, Amsterdam, Postbus 7057, 1007 MB, The Netherlands; Netherlands Institute for Health Services Research (Nivel), Utrecht, Postbus 1568, 3500 BN, The Netherlands

**Keywords:** natural language processing, activities of daily living, unstructured data, electronic health records, free-text notes

## Abstract

**Objective:**

Natural language processing (NLP) can enhance research on activities of daily living (ADL) by extracting structured information from unstructured electronic health records (EHRs) notes. This review aims to give insight into the state-of-the-art, usability, and performance of NLP systems to extract information on ADL from EHRs.

**Materials and Methods:**

A systematic review was conducted based on searches in Pubmed, Embase, Cinahl, Web of Science, and Scopus. Studies published between 2017 and 2022 were selected based on predefined eligibility criteria.

**Results:**

The review identified 22 studies. Most studies (65%) used NLP for classifying unstructured EHR data on 1 or 2 ADL. Deep learning, combined with a ruled-based method or machine learning, was the approach most commonly used. NLP systems varied widely in terms of the pre-processing and algorithms. Common performance evaluation methods were cross-validation and train/test datasets, with F1, precision, and sensitivity as the most frequently reported evaluation metrics. Most studies reported relativity high overall scores on the evaluation metrics.

**Discussion:**

NLP systems are valuable for the extraction of unstructured EHR data on ADL. However, comparing the performance of NLP systems is difficult due to the diversity of the studies and challenges related to the dataset, including restricted access to EHR data, inadequate documentation, lack of granularity, and small datasets.

**Conclusion:**

This systematic review indicates that NLP is promising for deriving information on ADL from unstructured EHR notes. However, what the best-performing NLP system is, depends on characteristics of the dataset, research question, and type of ADL.

## Introduction

The ever-increasing amount of data recorded by physicians or nursing staff in patients’ electronic health records (EHRs) offers opportunities for clinical practice and research. Although EHR systems are primarily designed for documentation about individual patient care, EHR data are increasingly used for scientific research. The data used for this purpose are predominantly structured data, which are recordings following a fixed format or category.

However, solely using structured EHR data for research could lead to biased results, for example, because this may lead to an underestimation of the incidence and prevalence,^1^^,^^2^ and low performance of prediction models^3^^,^^4^ of health problems.

Using unstructured health data, such as clinical notes and discharge letters, can enhance the quality of research results by capturing valuable information not found in structured data. It is estimated that more than half of all health records in the EHR systems are unstructured data.^5^ Even if health information could be recorded as structured data, healthcare professionals sometimes prefer to use unstructured free-text notes, for example, because they think it allows a more accurate representation of the patient’s situation.^6^^,^^7^

An example of health information often documented in both structured and unstructured manners, is the ability to perform a range of essential daily activities.^8–10^ The activities of ambulating, feeding, dressing, personal hygiene, continence, and toileting are referred to as activities of daily living (ADL).^11^ For care provision, adequate information on ADL is important for ensuring individuals receive the necessary daily support. Also, the research using health data on ADL requires adequate information to provide insight into the need for support with ADL and, for instance, the effect of a treatment on the ability to perform ADL.

ADL could be recorded in a structured way, using assessment tools such as the Barthel Index and Katz Activities of Daily Living Index.^12^ The International Classification of Functioning, Disability and Health (ICF) also categorizes different daily activities as part of a larger framework on functional status.^13^ Furthermore, there are ADL measures developed for a specific target population, for example, the Expanded Disability Status Scale (EDSS) (for Multiple Sclerosis)^14^ and the Karnofsky Performance Status Scale (for cancer).^15^ Despite the availability of assessment tools, ADL are also recorded in unstructured free-text notes in EHR systems.^8–10^

To extract information from unstructured EHR data, Natural Language Processing (NLP) is currently the most widely used “big data” analytical technique.^16^ NLP, a subfield of artificial intelligence, focuses on computers and human-language interaction. NLP can be used for various applications, such as information retrieval, text classification, topic identification, word frequency calculation, and sentiment analysis.^17^

Advancements in computing power, greater availability of large datasets, and recent breakthroughs in the field of NLP have increased the potential for generating valuable insights from unstructured EHR data.^18^ While the oldest NLP approach, the rule-based approach, relies on manual rule construction by experts, machine-learning approaches, including Support Vector Regression and Conditional Random Fields, are able to train algorithms with less manual coding.^19^ Rule-based and machine-learning models generally involve a pre-processing phase to standardize text by cleaning and preparing textual data as tokens, and a modeling phase in which unstructured textual data is fed into a model. In these models, pre-processing is crucial because the performance of the models depends on the quality of the data fed into the model.^20–22^ The latest breakthrough in machine learning is the deep-learning approach. Examples of deep-learning models are Word2vec^23^ and transformers, such as Bidirectional Encoder Representations from Transformers (BERT).^24^ In NLP, deep-learning models take a holistic approach considering the entire context and relationships within the sentence rather than individual tokens. This enables deep-learning models to analyze complex patterns in texts.^25^^,^^26^ In addition, the holistic approach avoids extensive pre-processing of texts.^27^

Although the opportunities for NLP in the healthcare sector are recognized, the usability for clinical practice and research depends on how well a NLP system performs.^28^ For example, overfitting is a common concern with machine-learning models. Overfitting means that an algorithm aligns too closely with a specific dataset, limiting its application to future data. Various evaluation methods, including train/test datasets and cross-validation, can be used to explore the performance of the NLP system and identify issues such as overfitting.^28–30^

While previous systematic reviews have explored the processing of unstructured clinical notes (eg,^19^^,^^31–35^) none have specifically focused on ADL. This gap makes it hard to draw conclusions and recommendations for using NLP to derive information on ADL from unstructured EHR notes. It is of significance to understand recent developments specific to NLP in the ADL research field as this will help researchers to gain a broader understanding, and provide insight into methods and techniques supporting and promoting new developments in the field of ADL research.

### Objective and research questions

This systematic review aims to give insight into the state of the art and usability of NLP systems to extract information on ADL from EHRs. The specific review questions addressed are as follows:

1. Which NLP systems are used to extract information on ADL from routinely recorded unstructured free-text data in EHRs?
2. Which methods are used to evaluate the performance of these NLP systems in research?
3. How do the NLP systems perform with regard to extracting information on ADL from EHRs?

## Methods

### Design

The reporting of this systematic review was guided by the PRISMA (Preferred Reporting Items for Systematic Reviews and Meta-Analyses) statement.^36^

### Search strategy and information sources

In November 2022, a librarian, in consultation with the authors of this paper, conducted searches in the Pubmed, Embase, Cinahl, Web of Science, and Scopus databases, using predetermined search strategies (Supplementary Appendix 1). The searches initially included studies on ADL and NLP published between January 1, 2012 and November 4, 2022. However, due to the large number of references generated by this search and given the review’s focus on recent developments, it was decided to exclude studies published before 2017; therefore only studies published in 2017 or later were included. After removal of 1339 duplicates and removal of 403 references published before 2017, 1277 references were retrieved. Additionally, 4 potential references were identified by checking the reference lists of relevant literature reviews.

### Article selection

**Figure 1.**
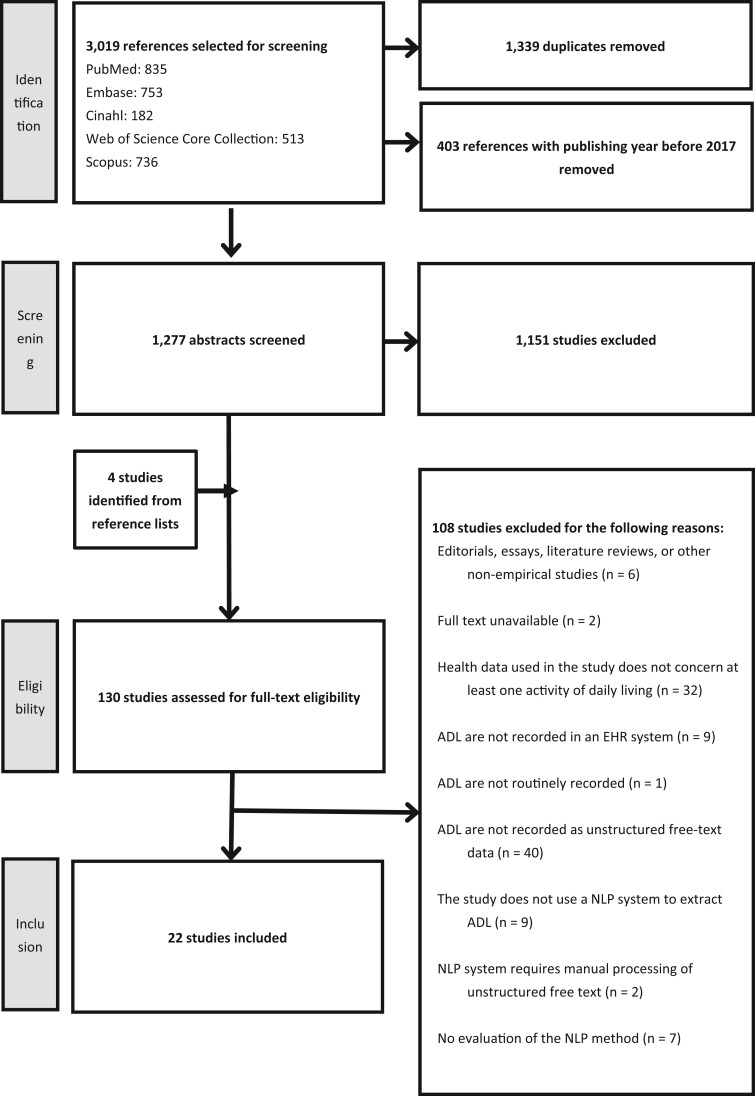
PRISMA (preferred reporting items for systematic reviews and meta-analyses) flow diagram.

Studies were selected in 2 steps, based on the eligibility criteria (see below). First, 2 reviewers independently reviewed the titles and abstracts of the 1277 studies (D.v.K. and Y.W.J.), resulting in a selection of 130 potentially relevant studies. Second, the full texts of these 130 studies were reviewed, in varying compositions, by 2 out of 4 reviewers (Y.W.J., D.v.K., Y.d.M., or M.O.V.). Discrepancies in selection were resolved through discussions between the reviewers (Y.W.J., D.v.K., Y.d.M., and M.O.V.). The steps and results of the selection process are described in the PRISMA flow diagram in Figure 1. After the final selection step, 22 studies were included for further analysis.

### Eligibility criteria

The following criteria were used by the reviewers during the selection of relevant studies.

1. *The study is an empirical study*
  All types of empirical studies were eligible for inclusion, including gray literature. Editorials, essays, literature reviews, or other non-empirical studies were excluded.
2. *The full text of the study had to be available*
3. *The study concerns at least 1 activity of daily living*
  Studies had to use information on at least 1 activity (ambulating, feeding, dressing, personal hygiene, continence, or toileting).^11^ There were no restrictions regarding care setting and age, health status, and type of disease of the study population.
4. *Study uses information on ADL that is routinely recorded as unstructured free text in an EHR system*
  To be included, studies had to use information on ADL that was recorded as unstructured free text in an EHR system by a healthcare professional. Studies were excluded if information on ADL was not routinely recorded, for example, if patients recorded them in a one-time questionnaire as part of scientific research.
5. *NLP system is used in the study to extract information on ADL*
  Studies were excluded if they only used manual processing of the unstructured free texts.
6. *The performance of the NLP system is evaluated and reported in the study*

#### Data extraction and synthesis

For each of the 22 included studies, 1 author (D.v.K. or Y.W.J.) manually extracted information, which was then verified by another author (Y.W.J., D.v.K., R.V., A.F., or M.O.V.). The extracted data were inserted in a prestructured format, developed in consultation with all the authors. The extracted information concerned background information on the study, including the aim of the study, country, whether the unstructured records were retrieved directly from an EHR system or other database, the study population, and type of ADL. Moreover, data were extracted on the NLP system, including the type (rule-based, machine learning, or deep learning), aim (eg, classification or data extraction), pre-processing steps, and tools. Lastly, data were extracted on the evaluation of the NLP system, including the metrics used to evaluate the NLP system, NLP system’s performance, and limitations of the method according to the authors of the specific study.

To address the research question regarding which NLP systems were used, we identified the most common aim (ie, data classification or extraction) and type of NLP system (rule-based, machine learning, deep learning, or a combination of these). To analyze trends in the NLP system used, we looked at whether the type of NLP system varied over the years. Furthermore, we determined frequently used pre-processing techniques and identified studies with no or few pre-processing steps. Lastly, we described the software used for the NLP. To answer the research question about which evaluation methods were used, we looked at the most commonly used methods and compared their prevalence across different types of NLP systems. To address the research question regarding the performance, we outlined the primary performance metrics used to evaluate the NLP system.

## Results

### Study characteristics

Twenty-two studies were included in the review (Table 1). The aim of the studies included is described in Table S1. The year with the most publications was 2019 (*n* = 6). Eleven studies were published in 2020 or later.

**Table 1.** Characteristics of the study and EHR data.

Study (year); country	Source(s) of routinely recorded EHRs on ADL	Study population	Type of ADL included
Anzaldi et al (2017)^37^; United States	Clinical notes from a nonprofit medical group (hospital, emergency department, and nursing home)	Patients aged over 65	Ambulating (walking difficulty) Continence (absence of fecal control and severe urinary control issues)
Kharrazi et al (2018)^2^^a^; United States	Same as^37^	Same as^37^	Same as^37^
Kan et al (2018)^38^^a^; United States	Same as^37^	Same as^37^	Same as^37^
Hernandez-Boussard et al (2017)^39^; United States	Clinical notes from a single, large, academic medical center	Prostate cancer patients	Continence (UI^a^)
Humbert-Droz et al (2022)^40^; United States	Rheumatology notes from the RISE^b^ registry	Rheumatology patients	Ambulating Feeding Dressing Personal hygiene Toileting
Alves et al (2022)^41^; United States	Clinical notes from neurology practices included in the OM1 MS Registry	Patients with MS^c^	Ambulating (mobility impairments)
Chen et al (2019)^42^; United States	Clinical notes from a large group practice	Patients aged over 65	Ambulating (walking difficulty) Continence (absence of fecal control and severe urinary control issues)
Banerjee et al (2019)^43^; United States	Clinical notes from a tertiary care academic Medical center included in a research database	Prostate cancer patients	Continence (UI^a^ and fecal incontinence)
Meskers et al (2022)^44^; The Netherlands	Clinical notes from a large teaching hospital	Hospitalized COVID-19 patients	Ambulating (mobility activities)
Rivera et al (2022)^45^; United States	Provider documentation, discharge notes, and PT and occupational therapy documentation from a large stroke referral center	Ischemic stroke patients	Ambulating (Modified Raking Scale)
Chen et al (2019)^46^; United States	Unstructured free-text from a large multispecialty medical group	Patients aged over 65	Ambulating (walking difficulty) Continence (absence of fecal control and severe urinary control issues)
Gori et al (2019)^47^; United States	Clinical notes from a tertiary academic medical center included in a research data warehouse	Prostate cancer patients	Continence (UI^a^)
Newman-Griffis et al (2018)^48^; United States	PT^d^ notes from a Clinical Center	Patients with PT^d^ notes	Ambulating (mobility)
Bozkurt et al (2020)^49^; United States	Clinical notes from an Academic Medical Centre included in a prostate cancer clinical data warehouse	Patients diagnosed with prostate cancer	Continence (UI^a^)
Doing-Harris et al (2019)^50^; United States	Clinical notes from a Veteran Health Administration Repository (VINCI)	Veterans diagnosed with cardiac disease	Ambulating (able to mobilize, bed-ridden, wheelchair-bound) Dressing
Goudar-zvand et al (2019)^51^; United States	Clinical notes and current visit information from the Mayo Clinic Biobank	Physician-diagnosed CI^e^ and CU^f^ patients aged 65 years and older	Ambulating (transferring) Feeding Dressing Personal hygiene (bathing) Toileting
Greve et al (2022)^52^; United States	Clinical notes from a single tertiary medical center	Patients with cerebral palsy	Ambulating
Thieu et al (2021)^53^; United States	PT^d^ notes from a Clinical Center	Patients with PTi notes	Ambulating (mobility domain of the ICF^g^)
Newman-Griffis et al (2021)^54^; United States	Notes from the Rehabilitation and Medicine Department at the NIH Clinical Center using databases of the NIH Biomedical Translational Research Information System	Patients receiving PT^d^	Ambulating (mobility activities)
Newman-Griffis et al (2021)^55^; United States	Claims database	Patients receiving disability benefits primarily related to musculo-skeletal, neurological, or mental impairments	Ambulating (mobility) Personal hygiene (self-care)
Sung et al (2021)^56^; Taiwan (English written records)	Clinical notes from 2 hospital stroke registries	Patients hospitalized for acute ischemic stroke	Ambulating (Modified Raking Scale)
Yang et al (2022)^57^; Canada	Clinical notes from a clinical database from a large MS^c^ clinic	Patients with MS^c^	Ambulating (mobility)

a UI: urinary incontinence.

b RISE: American College of Rheumatology’s Rheumatology Informatics System for Effectiveness.

c MS: Multiple Sclerosis.

d PT: Physical Therapy.

e CI: cognitive impaired.

f CU: Cognitive Unimpaired.

g ICF: International Classification of Functioning, Disability and Health.

Three studies were found to use the same dataset and NLP system.^2^^,^^37^^,^^38^ As 2 of them^2^^,^^38^ used the NLP system developed in the study by Anzaldi et al,^37^ we only refer to the study of Anzaldi et al^37^ in the remainder of this review. Thus, the total number of NLP systems we report on is 20.

### Data source

Of the 20 studies, 1 study used clinical notes written in Dutch,^44^ while the remaining 19 studies used English clinical notes^37^^,^^39–43^^,^^45–57^ (Table 1). Most clinical notes used were retrieved directly from an EHR system (*n* = 11).^37^^,^^39^^,^^42–46^^,^^48^^,^^52^^,^^53^^,^^57^ In the other studies, clinical notes were first transferred from an EHR system to a research database, registry, or claims database (*n* = 9).^40^^,^^41^^,^^47^^,^^49–51^^,^^54–56^ In such a database or registry, EHR data may be cleaned or combined with data from other sources before the data are transferred to the researchers.^58^^,^^59^

### Study population

The studies included in this review focused on a variety of diagnoses or patient groups (Table 1). The most frequently studied patient groups were patients aged over 65 (*n* = 4),^37^^,^^42^^,^^46^^,^^51^ patients with prostate cancer (*n* = 4),^39^^,^^43^^,^^47^^,^^49^ patients suffering from a chronic disease (*n* = 4),^40^^,^^41^^,^^52^^,^^57^ and patients receiving physical therapy (*n* = 3).^48^^,^^53^^,^^54^

### Activities of daily living

Each of the 6 ADL is covered in at least 1 study (Table 1). However, none of the studies covered all 6 ADL. The majority of studies focused on 1 activity (*n* = 13),^39^^,^^41^^,^^43–45^^,^^47–49^^,^^52–54^^,^^56^^,^^57^ while others covered 2 (*n* = 5)^37^^,^^42^^,^^46^^,^^50^^,^^55^ or 5 (*n* = 2)^40^^,^^51^ activities. The most frequently studied activities were ambulating (*n* = 16)^40–42^^,^^44–46^^,^^48^^,^^50–57^^,^^60^ and continence (*n* = 7).^37^^,^^39^^,^^42^^,^^43^^,^^46^^,^^47^^,^^49^

### Purpose of using NLP

In 70% of the studies, NLP was used for classification purposes (*n* = 14/20),^37^^,^^42–46^^,^^49^^,^^50^^,^^52^^,^^54–57^ for example, classifying patients as frail or not,^46^^,^^50^ determining the presence and severity of urine incontinence,^43^^,^^49^ and assigning ICF categories.^54^^,^^55^ The remaining studies used NLP for information extraction (*n* = 3),^39–41^ for Named Entity Recognition (*n* = 2),^48^^,^^53^ or topic modeling (*n* = 1),^51^ as is shown in Table S2.

### Type of NLP

The rule-based approach, the oldest and simplest NLP approach, was used as the sole method in 3 studies,^37^^,^^39^^,^^40^ while another study combined rule-based with deep learning (Table S2).^49^

More than half of the NLP systems relied on machine learning (*n* = 12); all of these studies were published in 2019 or later.^41–45^^,^^50–56^ Eight of these studies applied a combination of machine learning and deep learning.^44^^,^^50–56^^,^^60^ Various machine-learning algorithms were used, with Support Vector Machines (SVMs) being the most prevalent (*n* = 5).^44^^,^^50^^,^^52^^,^^54^^,^^55^

Thirteen studies applied deep learning; all of them were published in 2018 or later.^44^^,^^46–57^ Word2Vec was used in 7 studies.^46–49^^,^^52^^,^^54^^,^^57^ Among the 13 deep-learning NLP systems, 2 studies were based on ClinicalBERT^55^^,^^56^ and 1 study used BERTje.^44^ These 3 studies were published in 2021 or later.

### Pre-processing

Table 2 shows a variety of pre-processing steps applied to prepare unstructured notes, with tokenization the most frequently used pre-processing technique (*n* = 10).^39–42^^,^^46^^,^^49^^,^^52–55^

**Table 2.** Pre-processing steps used in the included studies.

Pre-processing step	Number of studies	References
Tokenization	10	^39–42^ ^,^ ^46^ ^,^ ^49^ ^,^ ^52–55^
Stop-word removal	6	^41^ ^,^ ^43^ ^,^ ^49^ ^,^ ^51^ ^,^ ^56^ ^,^ ^57^
None	4	^37^ ^,^ ^44^ ^,^ ^45^ ^,^ ^48^
Normalization	3	^43^ ^,^ ^55^ ^,^ ^56^
Removal of redundant information	3	^40^ ^,^ ^49^ ^,^ ^57^
Sentence splitting	2	^39^ ^,^ ^49^
Lemmatization	2	^41^ ^,^ ^52^
Stemming	2	^43^ ^,^ ^51^
Sentence segmentation	2	^42^ ^,^ ^54^
Lowercase	2	^55^ ^,^ ^56^
Removal of identifying information	2	^40^ ^,^ ^57^
Standard tool for text-cleaning methodologies, not further defined	1	^47^
Manual	1	^50^
Removal of formatting	1	^40^

In general, deep learning requires less pre-processing compared to rule-based and machine-learning models. In the 3 studies that employed deep learning only, little to no pre-processing was performed. One of the 3 studies^46^ only used sentence segmentation, the second study reported no pre-processing steps,^48^ and the third study used a standard tool for text-cleaning methodologies.^47^ However, as the precise methodologies applied to the dataset were not specified in this last study, the extent of data pre-processing remains unclear.

Pre-processing details were not reported in 3 other studies. Anzaldi et al used a rule-based approach for identifying geriatrics syndromes in EHR free-text notes as well as the explicit mention of “frailty” in the notes.^37^ In such an approach, pre-processing is not always a necessity. In the study of Rivera et al, although pre-processing was not reported, we cannot conclude that data pre-processing was therefore not applied as a machine learning usually involves pre-processing.^45^ Lastly, Meskers et al used BERTje to create vectors instead of pre-processing methods.^44^

### Software

The studies used different software (Table S2). Most studies used Python (*n* = 11).^40^^,^^41^^,^^43^^,^^44^^,^^49^^,^^50^^,^^53–57^ For 4 studies it is unclear which software was used.^37^^,^^47^^,^^48^^,^^52^ In addition, Javascript or a tool developed for NLP applications, including cTakes, GATE, MedTagger, and CRFSuite were used.^39^^,^^42^^,^^45^^,^^46^^,^^51^

### Methods used to evaluate NLP system performance

Almost all studies used cross-validation or train-/test datasets to evaluate the NLP system’s performance (Table S2). Six studies evaluated their NLP system using both cross-validation and train-/test datasets.^43^^,^^44^^,^^49^^,^^52^^,^^55^^,^^57^ In 4 studies, an expert manually evaluated the performance of the NLP system. Five other studies used solely train-/test datasets (*n* = 5) and 4 other studies only cross-validation (*n* = 4).^50^^,^^53^^,^^54^^,^^56^

The study by Goudarzvand et al used recent publications to evaluate their NLP system,^51^ which was used for topic modeling—the only study in our review with this purpose. As topic modeling lacks a gold standard for comparing the outcome of the model with, they validated the results against recent publications to verify whether meaningful outcomes were generated.

The 3 most frequently reported evaluation metrics for the NLP performance for ADL were the F1 score (or harmonic mean of precision and sensitivity), (*n* = 12), precision (or positive predictive value) (*n* = 8), and sensitivity (or recall) (*n* = 7). Other primary evaluation metrics reported were accuracy (*n* = 4), area under the curve (AUC) (*n* = 4), Inter-Annotator Agreement (*n* = 3), specificity (*n* = 1), negative predictive value (*n* = 1), false positive rate (*n* = 1), and root-mean-square error (*n* = 1).

### Outcomes of the performance evaluation

More than half of the studies reported relatively high scores for the evaluation metrics (*n* = 12),^37^^,^^39^^,^^41^^,^^44^^,^^47–50^^,^^52–54^^,^^56^ indicating good performance by the NLP systems for that dataset and the purpose of the study (Table S2). This was particularly the case for systems extracting information on ambulating.

Other studies reported mixed performance outcomes (*n* = 7).^40^^,^^42^^,^^43^^,^^45^^,^^46^^,^^55^^,^^57^ Some of the studies showed different outcomes when the results were stratified based on the type of ADL. For instance, Chen et al used a machine-learning model for classifying geriatric syndrome constructs. High scores were obtained for fecal control (F1 = 0.857) and walking difficulty (F1 = 0.758), but for severe urinary control issues low scores were obtained (F1 = 0.532).^42^ Another study by Chen et al applied a deep-learning model for the same constructs and obtained comparably mixed scores.^46^ Humbert-Droz et al found that scores varied depending on the method used to evaluate the NLP system. They evaluated NLP performance by comparing the outcome of the NLP tool with (1) a manual review, (2) structured EHR data, and (3) an external database. The highest scores for sensitivity, positive predictive value, and F1 scores were observed for the manual review, while the lowest scores were found in the comparison with structured EHR data. Humbert-Droz et al pointed out that this does not necessarily reflect the NLP system’s performance. They encountered several issues with structured EHR data limiting their use as the gold standard in an evaluation.^40^ Furthermore, mixed results were found when different approaches were compared. For example, Yang et al showed higher scores for a combined ruled-based and deep-learning model, compared to the scores for each approach individually. They noted that this hybrid approach was better at leveraging the strengths of each approach and tackling challenges with regard to the dataset, including imbalanced data.^57^

Authors of the studies in this systematic review identified several limitations concerning the NLP systems they developed. Generalizability to other healthcare sectors, practices, languages, patient groups, or data sources emerged as a significant challenge,^13^^,^^37^^,^^39–41^^,^^43^^,^^47^^,^^49^^,^^52–57^ as the NLP systems were trained on datasets with specific characteristics. Another major challenge relates to the dataset on which the NLP system is trained and tested. Authors reported issues with small datasets due to factors such as restricted access to relevant EHR data, few amount of notes per patient due to a short hospital stay, or few patients in the study sample.^37^^,^^39^^,^^49^^,^^54^ In addition, inadequate documentation and lack of granularity were mentioned.^41^^,^^43–45^^,^^49^^,^^51^^,^^52^^,^^54^^,^^57^

## Discussion

### Principal findings

This systematic review provides a comprehensive overview of current research employing NLP to extract information on ADL from unstructured free-text notes in EHRs. Adequate information on ADL is important for care provision and research to ensure that individuals receive the necessary daily support. As information on ADL is often recorded in unstructured free-text EHR notes, NLP could be valuable for deriving this information. We explored 20 NLP systems described in 22 studies. Most studies (65%) utilized NLP for classifying unstructured EHR data on 1 or 2 ADL. Our findings show that a variety of NLP methods, algorithms, and pre-processing steps were used. There was a notable prevalence of deep-learning approaches. The majority of studies using deep learning also applied ruled-based methods or machine learning. Evaluation of the NLP system’s performance predominantly involved train-/test datasets and cross-validation. The studies included in this review used a wide range of evaluation metrics, including F1, precision, and sensitivity. Despite the variety of NLP approaches and evaluation metrics, most studies reported relativity high overall scores on the evaluation metrics, indicating that the characteristics of the best-performing NLP system depend on study-specific factors.

The variability in models, approaches, and reporting complicates the direct comparison between the NLP systems and the quest for the best possible method. However, overall, the results of this review indicate that NLP systems are promising for research using unstructured EHR data on ADL for the following reasons.

First, the field of NLP is developing rapidly. It has evolved from ruled-based methods to machine learning and deep learning. Compared to previous systematic reviews on the use of NLP for unstructured EHR notes, we included relatively more deep-learning approaches.^32^^,^^34^^,^^35^ This shows that relatively new deep-learning algorithms, including transformers such as BERT, are being studied for NLP systems to extract information from unstructured clinical notes on ADL.

To improve the performance of the NLP system, often multiple approaches are compared or combined. Most studies adopted a hybrid approach by combining deep learning with ruled-based or machine-learning algorithms in their final model. The possible benefits of hybrid approaches are also recognized by systematic reviews that focused on the application of NLP in other healthcare domains, including radiology,^61^^,^^62^ clinical information in general,^31^^,^^34^ and chronic diseases.^35^ Hybrid approaches may be better able to address challenges related to the dataset, such as small or imbalanced datasets. Some of the studies included in this review encountered challenges with the datasets arising from how the information was recorded during healthcare provision, such as inadequate recordings or a low level of granularity, or because they did not have access to all relevant EHR data. These challenges are not unique to unstructured data but are also mentioned in the broader literature discussing data quality challenges in the use of EHR data for research (eg,^59^^,^^63^)

Second, the characteristics of the best-performing NLP system depend on the context in which the dataset is generated, such as different EHR systems and different healthcare organizations. The studies included in this review that retrieved the data directly from an EHR system, rather than from a research database or registry, had access to data from a single organization or from organizations belonging to one medical group. It is expected that the NLP system will perform differently on datasets with other characteristics. NLP systems trained on datasets from multiple sources with different characteristics will have a higher external validity.

Third, a variety of metrics were used to evaluate the performance of the NLP systems. However, most studies evaluated the performance with train-/test datasets and cross-validation and reported F1 scores. Although the most appropriate evaluation metrics depend on the research aim, F1 scores are commonly valuable in many cases, especially for classification purposes, which was the most prevalent purpose of the NLP systems in this review. Almost all F1 scores exceeded 0.7. This indicates that the methodologies used in developing the NLP systems, considering the characteristics of the specific dataset and research question of the study, are promising for generating information on ADL from unstructured EHR data.

#### Strengths, limitations, and recommendations for further research

To the best of our knowledge, this is the first systematic review exploring NLP systems for extracting information on ADL from unstructured EHR data. A strength is that we used a broad search strategy in 5 different literature databases. However, the following limitations should be kept in mind. First, while ambulating and continence were covered by most studies, some ADL were only included in a few NLP systems. More research on NLP systems covering all 6 ADL is recommended. Second, some studies provided limited information on the algorithms, for example with few details on the pre-processing. Future NLP studies should prioritize adequate reporting, as is emphasized in other systematic reviews as well.^61^^,^^64–66^ Third, the field of NLP is developing rapidly. To keep up with the developments, it is recommended to conduct the search again in the near future.

## Conclusion

The results of this systematic review indicate that NLP is a promising method for deriving information on ADL from unstructured EHR notes. Various NLP systems are already used in research and show overall good evaluation outcomes. Choosing which NLP system will perform best, depends on the characteristics of the dataset, research question, and type of ADL studied. Since there is no one-size-fits-all method, our findings suggest that research on ADL could benefit from an iterative process in which different NLP approaches are compared or combined based on the performance evaluation outcomes. Future developments in NLP for ADL extraction should focus on addressing generalizability issues and refining evaluation methodologies.

## Supplementary Material

ooae044_Supplementary_Data

## Data Availability

Data are available on request.
