# Supplementary material for: Natural language processing systems for extracting information from electronic health records about activities of daily living. A systematic review
Source: JAMIA Open. 2024 May 24;7(2):ooae044. doi: 10.1093/jamiaopen/ooae044 (PMC11126158; doi:10.1093/jamiaopen/ooae044)
Supplement: ooae044_Supplementary_Data [file ooae044_supplementary_data.zip › Supplementary Table 2_revised version.docx]

Supplementary Table 2. Information about the NLP system and evaluation of the performance.

| **Study** | **Pre-processing steps** | **NLP approach** | **Purpose of NLP use** | **Classification** | **Tool used, including libraries** | **Method to evaluate NLP performance** | **Primary evaluation metrics and performance scores with regard to ADL** |
| --- | --- | --- | --- | --- | --- | --- | --- |
| Anzaldi et al. (2017) [37] | n/a | - Rule-based (Regex) | Text classification | Ten geriatric syndromes | n/a | Manual review of subset | - False positive rates for the syndromes ranged from 1% to 15%. - Frailty: a manual review of 164 random samples revealed a false positive rate of 3%. |
| Kharrazi et al. (2018) [2]^k^ | Same as [37] | Same as [37] | Same as [37] | Same as [37] | Same as [37] | Same as [37] | Same as [37] |
| Kan et al. (2018) [38]^k^ | Same as [37] | Same as [37] | Data extraction | n/a | SAS | Same as [37] | Same as [37] |
| Hernandez-Boussard et al. (2017) [39] | - Sentence splitting - Tokenization | - Rule-based (Regex) | Data extraction | n/a | GATE software | Manual review of subset | - F1 score: 87% affirmed, 96% negated, 91% discuss risk. - Precision: 87% affirmed, 94% negated, 92% discuss risk. - Recall: 87% affirmed, 97% negated, 90% discuss risk. |
| Humbert-Droz et al. (2022) [40] | - Annotate concepts, and extract associated numerical scores - Non-numerical score resolution, and numerical score cleaning - Remove formatting and deidentification of notes - Tokenization | - Rule-based (Regex) | Data extraction | n/a | Python with the libraries BeautifulSoup and Spacy | Manual review of subset | - Manual chart review Sensitivity: 95%, PPV^a^: 87%, and F1 score: 91%. - Agreement between structured and unstructured documented scores  DA: κ = 0.43–0.68  FS measures: κ = 0.86–0.98 - Manual chart review to compare scores documented in notes with structured data  Sensitivity: 39%, PPV: 73%, F1 score: 51%. - External validation Sensitivity: 92%, PPV: 69%, F1 score: 79% - Overall accuracy of the NLP system: 86% |
| Alves et al. (2022) [41] | - Lemmatization - Stop-word removal - Tokenization | - Machine learning (XGBoost regression) | Data extraction | n/a | Python language using the NLTK^b^ package | Train-/test dataset | Evaluation of performance using the binarized version of the outcome in the validation cohort:   - PPV: 0.85 - NPV^c^: 0.85 - AUC^d^: 0.91 |
| Chen et al. (2019) [42] | - Sentence segmentation - Tokenization | - Machine learning (CRF) | Text classification | Ten geriatric syndrome constructs | - cTAKEs - CRFSuite software package | Test dataset evaluation | Final CRF model performed at patient-level determination   - Macroaverage F1: 0.834, microaverage F1: 0.851 - However, performance varied by construct.   Phrase-partial evaluation of the CRF model:   - Absence of fecal control  F1 score: 0.857, precision: 1, recall: 0.750 - Severe urinary control issues  F1 score: 0.532, precision: 0.689, recall: 0.433 - Walking difficulty:  F1 score: 0.758, precision: 0.842, recall: 0.689 |
| Banerjee et al. (2019) [43] | - Number-to-string conversion - Statistical term expansion techniques - Stemming - Stop word removal | - Machine learning (multinomial logistic regression, Naive Bayes for multinomial Bernoulli models) | Text classification | UI^f^ and BD^g^: Presence, absence, or risk discussion | NLTK package in Python | - 5-fold cross-validation - Train-/test dataset | - Both UI and BD  Average F1 score: 0.86, precision: 0.88, recall: 0.85 - Neural embedding model Ability to classify correctly 80% UI cases and 91% BD. - Doc2vec:  UI: Overall F1: 0.55  BD: Overall F1: 0.62 - Tf-idf weighted embedding method Both UI and BD: F1 score: 0.86 - Note Level Annotation:  F1 score: 0.9 |
| Meskers et al. (2022) [44] | n/a | - Machine learning  (SVM^h^ for the multilabel classifier and logistic regression model for level labelling) - Deep learning (BERTje) | Text classification | COVID-19 relevant ICF^i^ categories and levels (Walking and moving, exercise tolerance, emotional functions, work and employment) | Python by using Scikit-learn | - 5-fold cross-validation - Train-/test dataset | ICF classifier (constructed by SVM, logistic regression and BERTje)   - Average Inter-Annotator Agreement: 0.81 - Walking and Moving:   F1 score: 0.7 |
| Rivera et al. (2022) [45] | n/a (unknown) | - Machine learning (Naive Bayes) | Text classification | Modified Rankin Scale | An open-source naive Bayes classifier written in JavaScript | - Model 1: n/a. - Model 2: Train-/test dataset | Validation set to validate predictions against known mRS values:   - 73-percentage agreement with vascular neurology assessment - Average mRS score difference of e0.28 - Absolute average difference of 1.22 on the 7-point scale for those that were incorrect. |
| Chen et al. (2019) [46] | - Sentence segmentation | - Deep learning (Word2Vec with LSTM^j^) | Text classification | Ten geriatric syndromes | cTAKES | Test set evaluation | Micro-F1 of best-performing model:   - At sentence level: 0.605 - At patient level: 0.843   F1 scores of the best-performing models by construct at sentence and patient level:   - Absence of fecal control:  Sentence: 0.857 / patient: 0.800 - Severe urinary control issues:  Sentence: 0.495 / Patient: 0.571 - Walking difficulty:   Sentence: 0.642 / Patient: 0.879.   - In addition, precision and recall scores were determined. |
| Gori et al. (2019) [47] | Standard tool of text cleaning methodologies (e.g. stop words and punctuation removal, number to string conversion, and bigram formation using mutual information) | - Deep learning (neural word embedding, Word2vec) | Text classification | If someone is continent/incontinent | n/a | Manual review of subset | UI annotation: F1 score: 0.86 on 200 sentences extracted from the clinical notes. |
| Newman-Griffis et al. (2018) [48] | n/a | - Deep Learning (LSTM model using Word2Vec3 and FastText4) | Named Entity Recognition | n/a | n/a | Test set evaluation | Embeddings trained on a small in-domain corpus perform nearly as well as those learned from large out-of-domain corpora, and domain adaptation techniques yield additional improvements in both precision and recall. In addition, F1 scores were determined. |
| Bozkurt et al. (2020) [49] | - Numbers and punctuation removal - Removal of extra white spaces - Sentence splitting - Stop word removal - Tokenizer | - Rule-Based (Regex) - Deep learning (Convolutional Neural Network with Word2Vec) | Text classification | Classification of positive UI cases: mild, moderate, or severe | NLTK package in Python | - 5-fold cross-validation - Train-/test dataset | Overall accuracy disease severity categories:   - Rule-based model: 0.86 - Deep-learning model: 0.73   Mild and moderate groups in the deep learning model:   - Recall rates: 0.78 - Precision rate: 0.79 - A hybrid model that combined both methods did not improve the accuracy of the rule-based model but did outperform the deep learning model (accuracy: 0.75). |
| Doing-Harris et al. (2019) [50] | Manual review of snippets | - Machine learning (SVM) - Deep learning (Deep Neural Network) | System 1: Classification   System 2: prediction of patient mortality | Frailty or not | Python by using Scikit-learn | 10-fold cross-validation | - SVM model average accuracy score: 80.5% - DNN model AUC: 78.3% (95% CI 77.1 to 79.5%) |
| Goudar-zvand et al. (2019) [51] | - Stemming - Stop word removal | - Machine learning (TKM) - Deep learning (KATE) | Topic modelling | n/a | MedTaggerIE module in MedTagger | Validated by recent publications | - Validation based on aggregated term frequencies. Results were visualized to show the hidden potential topics that may contribute to developing CI. These results were validated by recent publications and showed promising outcomes. However, some common topic words, not relevant to CI but may appear in any diseases, were also captured. - Overall, the recent models TKM and KATE were better at capturing the semantically meaningful representation of the data compared to LDA. - KATE model generated more words related to CI than the TKM model. |
| Greve et al. (2022) [52] | - Lemmatization - Tokenization | - Machine learning (Logistic regression and SVM) - Deep learning (Word2vec) | Text classification | Ambulatory status and GMFCS levels. | n/a | - 10-fold cross-validation - Train-/test dataset | NLP (logistic regression) applied to the EHR differentiated between:   - GMFCS levels I–II and III:  15% sensitivity, 96% specificity, 46% PPV, 0.71 AUC - GMFCS levels IV and V: 81% sensitivity, 51% specificity, 70% PPV, 0.75 AUC. |
| Thieu et al. (2021) [53] | - Tokenization | - Machine learning (CRF) - Deep learning (Recurrent neural network and BERT) | Named Entity Recognition | n/a | - Stanford CoreNLP - Python by using Scikit-learn | 5-fold cross-validation | Average F1 score on exact entity matching of our Ensemble method (84.90 %) outperformed popular NER methods:   - Conditional Random Field (80.4 %) - Recurrent Neural Network (81.82 %) - Bidirectional Encoder Representations from Transformers (82.33 %) |
| Newman-Griffis et al. (2021) [54] | - Sentence segmentation - Tokenization | - Machine learning (Word2Vec, KNN and linear-kernel SVM) - Deep learning (feed-forward Deep Neural Network) | Text classification | ICF | Python by using Spacy and Wordpiece | 10-fold cross-validation | Performance for coding FSI in the mobility domain for the best performing model (SVM with PT-OT embedding features): macro-averaged F1 84.0% |
| Newman-Griffis et al. (2021) [55] | - Lowercase - Normalization of numbers, URLs, dates, and times - Tokenization | - Machine learning (SVM) - Deep learning approach (ClinicalBert) | Text classification | ICF categories to mentions of functional activity | Python by using Spacy | - 10-fold cross-validation - Train-/test dataset | Mobility dataset:   - With Action oracle:  F1 score: 0.696 (for both NIHCC and SSA) - Without Action oracle:  NIHCC F1 score :0.553; SSA F1: 0.541, p-value: 0.9. - NIHCC embeddings were statistically significantly better than the next best clinicalBERT features (F1 of 0.553 vs. 0.531; p-value = 0.025) without the Action oracle - SSA embeddings were not significantly different from clinicalBERT (F1 of 0.541 vs 0.531; p-value = 0.17). |
| Sung et al. (2021) [56] | - Acronyms and abbreviations were expanded to their full forms - Lemmatization - Lowercase - Removal of non-ASCII characters and nonword special characters - Stop word Removal - Words were spell-checked and corrected | - Machine learning (Random Forest) - Deep learning (ClinicalBERT) | Text classification | Modified Ranking Scale | Python | 10-fold cross-validation | Model using history of present illness   - AUC internal sets: 0.820 - AUC external validation: 0.792 - Comparable to the National Institutes of Health Stroke Scale scores (0.811 and 0.807).   Model using computed tomography reports   - AUC internal sets: 0.758 - AUC external validation: 0.658. |
| Yang et al. (2022) [57] | - Removal of identifying information - Removal of redundant information - Stop word removal | - Rule-based - Deep learning (Word2Vec and Convolutional Neural Network) | Text classification | Total EDSS score and EDSS functional system subscores | - Python by using NLTK - Gensim (Word2Vec) | - 10-fold cross-validation - Train-/test dataset | - Combined keyword-CNN model: Accuracy: 0.90, precision: 0.83, recall: 0.83, F-score: 0.83 - Rule-based model:  Accuracy: 0.57, precision: 0.91, recall: 0.65, F-score: 0.70 - CNN model:  Accuracy: 0.86, precision: 0.70, recall: 0.70, F-score: 0.70 |

^a^PPV: Positive Predictive Value ^i^ICF: International Classification of Functioning, Disability and Health

^b^NLKT: Natural Language Toolkit ^h^SVM: Support Vector Machine

^c^NPV: Negative Predictive Value ^j^LSTM: Long short-term memory

^d^AUC: Area Under the Curve ^k^ As these studies [2, 38] used the NLP system developed in the study by Anzaldi et al (2017) [37], we only refer

^e^CRF: Conditional Random Field to the study of Anzaldi et al. [37] in the review.

^f^UI: urinary incontinence

^g^BD: bowel dysfunction
