# Supplementary material for: Natural language processing systems for extracting information from electronic health records about activities of daily living. A systematic review
Source: JAMIA Open. 2024 May 24;7(2):ooae044. doi: 10.1093/jamiaopen/ooae044 (PMC11126158; doi:10.1093/jamiaopen/ooae044)
Supplement: ooae044_Supplementary_Data [file ooae044_supplementary_data.zip › Supplementary Table 1_revised version.docx]

Supplementary Table 1. Characteristics of the study and EHR data.

| **Study (year); country** | **Aim/scope of study according to authors** |
| --- | --- |
| Anzaldi et al. (2017) [37]; United States | To determine which geriatric syndromes were most associated with provider descriptions of frailty, and how patients described as “frail” differed from other older adults in terms of geriatric syndrome burden and healthcare utilization. |
| Kharrazi et al. (2018) [2]; United States | To examine the value of unstructured electronic health record data (free-text notes) in identifying a set of geriatric syndromes. |
| Kan et al. (2018) [38]; United States | To (1) define and compare the prevalence of geriatric risk factors among older adults based on information available from claims, structured EHRs, and unstructured EHRs; and (2) estimate the relationship between geriatric risk factors and health care utilization. |
| Hernandez-Boussard et al. (2017) [39]; United States | To develop and validate an NLP pipeline to detect important patient-centered outcomes as interpreted and documented by clinicians in their dictated notes. |
| Humbert-Droz et al. (2022) [40]; United States | To develop and evaluate an NLP pipeline for extracting disease activity and functional status assessment scores. |
| Alves et al. (2022) [41]; United States | To validate a machine learning model to estimate EDSS^a^ scores. |
| Chen et al. (2019) [42]; United States | To automatically determine if a patient has any geriatric syndromes by mining the free texts of associated EHR clinical notes and to assess which statistical NLP techniques are most effective. |
| Banerjee et al. (2019) [43]; United States | To present and demonstrate the accuracy of an NLP pipeline that targets to assess the presence, absence, or risk discussion of UI^b^ and BD^c^ following prostate cancer treatment. |
| Meskers et al. (2022) [44]; The Netherlands | To address the feasibility, reliability, and internal validity of NLP for automated functional assessment of hospitalized COVID-19 patients in key ICF^d^ categories and levels from unstructured text in EHRs. |
| Rivera et al. (2022) [45]; United States | To describe the outcomes from treatments for ischemic stroke alone and in combination with a tertiary referral center experience. |
| Chen et al. (2019) [46]; United States | To automatically identify vulnerable older adult patients with geriatric syndrome based on clinical notes extracted from an EHR system, and demonstrate how contextual information can improve the process. |
| Gori et al. (2019) [47]; United States | To assess documentation of UI^b^ in prostatectomy patients using unstructured clinical notes from EHRs. |
| Newman-Griffis et al. (2018) [48]; United States | To present the first analysis of automatically extracting descriptions of patient mobility. |
| Bozkurt et al. (2020) [49]; United States | To propose an NLP pipeline to identify the severity of UI^b^ in prostate cancer patients using only free-text clinical notes from EHRs. |
| Doing-Harris et al. (2019) [50]; United States | To create a machine-interoperable description of frailty that reflects all the areas that clinicians consider when deciding which cardiac intervention will best serve the patient as well as general indications of frailty found in patient records. |
| Goudar-zvand et al. (2019) [51]; United States | To characterize and better understand early signals of elderly patient CI^e^ by examining temporal trends of patient ADL and analyzing topics of patient medical conditions in clinical free text using topic models. |
| Greve et al. (2022) [52]; United States | To predict ambulatory status and GMFCS^g^ levels in patients with cerebral palsy by applying NLP EHR clinical notes. |
| Thieu et al. (2021) [53]; United States | To set a foundational step to utilize mobility information in clinical NLP. |
| Newman-Griffis et al. (2021) [54]; United States | To present a general-purpose approach to expanding NLP technologies to assign standardized codes to new types of information in the EHR, and apply this approach to produce new technologies for linking EHR text to the ICF^d^. |
| Newman-Griffis et al. (2021) [55]; United States | To investigate NLP methods for automatically coding documentation of key domains of functioning to the ICF^d^ and evaluate their performance on coding medical records associated with claims for federal disability benefits submitted to the U.S. Social Security Administration. |
| Sung et al. (2021) [56]; Taiwan (English written records) | To test whether using unstructured text in electronic health records can improve the prediction of functional outcomes after acute ischemic stroke. |
| Yang et al. (2022) [57]; Canada | To compare rule-based and deep-learning NLP algorithms for detecting and predicting the total EDSS^a^ score and EDSS functional system subscores |
