# Supplementary material for: Natural language processing systems for extracting information from electronic health records about activities of daily living. A systematic review
Source: JAMIA Open. 2024 May 24;7(2):ooae044. doi: 10.1093/jamiaopen/ooae044 (PMC11126158; doi:10.1093/jamiaopen/ooae044)
Supplement: ooae044_Supplementary_Data [file ooae044_supplementary_data.zip › Appendix Search Histories 4 november 2022.docx]

# Appendix 1

**PubMed History and Search Details November 4, 2022**

| **Search** | **Query** | **Results** |
| --- | --- | --- |
| #5 | #4 AND 2012/01/01:2023/12/01[dp] | 835 |
| #4 | #1 AND #2 AND #3 | 1,081 |
| #3 | "Activities of Daily Living"[Mesh] OR "Functional Status"[Mesh] OR "Mobility Limitation"[Mesh] OR "Locomotion"[Mesh:NoExp] OR "Walking"[Mesh] OR "Stair Climbing"[Mesh] OR "Hygiene"[Mesh] OR "Urinary Incontinence"[Mesh] OR ADL[tiab] OR “activities of daily living*”[tiab] OR “daily living activit*”[tiab] OR “daily physical activ*”[tiab] OR “activity limitation*”[tiab] OR “limitation of activit*”[tiab] OR “limitation of daily activ*”[tiab] OR “independent living*”[tiab] OR “everyday function*”[tiab] OR ”functional abilit*”[tiab] OR “functional able*”[tiab] OR “functional status”[tiab] OR “patient functioning”[tiab] OR “physical abilit*”[tiab] OR “physical able*”[tiab] OR disability[tiab] OR “daily function*”[tiab] OR “mobility”[tiab] OR “ambulating”[tiab] OR ambulation[tiab] OR self-care[tiab] OR selfcare[tiab] OR eating[tiab] OR feeding[tiab] OR drinking[tiab] OR bathing[tiab] OR shower*[tiab] OR wash*[tiab] OR “personal care”[tiab] OR “personal hygien*”[tiab] OR cleanliness[tiab] OR “oral hygiene”[tiab] OR toothbrushing[tiab] OR “getting dressed”[tiab] OR “getting undressed”[tiab] OR “dressing”[tiab] OR walking[tiab] OR “stair climbing”[tiab] OR “stairs climbing”[tiab] OR “climbing stairs”[tiab] OR grooming[tiab] OR toileting[tiab] OR “toilet use”[tiab] OR “going to the toilet”[tiab] OR excretion[tiab] OR “urine control*”[tiab] OR “bowel control*”[tiab] OR “controlling bowel*”[tiab] OR “bladder control*”[tiab] OR “controlling bladder”[tiab] OR continenc*[tiab] OR incontinenc*[tiab] | 1,514,009 |
| #2 | "Routinely Collected Health Data"[Mesh] OR "Medical Records"[Mesh] OR "Medical Records Systems, Computerized"[Mesh] OR “routinely record*”[tiab] OR “routinely collect*”[tiab] OR “medical record*”[tiab] OR “health record*”[tiab] OR EHR[tiab] OR EHRs[tiab] OR EMR[tiab] OR EMRs[tiab] OR “patient record*”[tiab] OR “health data”[tiab] OR “routine data”[tiab] OR “administrative data”[tiab] OR “clinical data”[tiab] OR “real world data”[tiab] OR “health information exchang*”[tiab] OR “medical information exchang*”[tiab] OR “health summar*”[tiab] OR "medical histor*"[tiab] OR "patient histor*"[tiab] OR "patient coding*"[tiab] | 485,546 |
| #1 | "Natural Language Processing"[Mesh] OR "Data Mining"[Mesh] OR "Information Storage and Retrieval"[Mesh] OR “natural language*”[tiab] OR NLP[tiab] OR “text mining”[tiab] OR textmining[tiab] OR “data mining”[tiab] OR datamining[tiab] OR “information retriev*”[tiab] OR “information extract*”[tiab] OR “data extract*”[tiab] OR “data retriev*”[tiab] OR “machine learning”[tiab] OR “deep learning”[tiab] OR “active learning”[tiab] OR “sentiment analys*”[tiab] OR “opinion mining*”[tiab] OR “sentiment classification*”[tiab] OR “unstructured text*”[tiab] OR “unstructured data”[tiab] OR free-text*[tiab] OR freetext*[tiab] | 369,935 |

**Embase History and Search Details November 4, 2022**

| **Search** | **Query** | **Results** |
| --- | --- | --- |
| #6 | #5 NOT 'conference abstract'/it | 753 |
| #5 | #1 AND #2 AND #3 AND [2012-2022]/py | 1,220 |
| #4 | #1 AND #2 AND #3 | 1,405 |
| #3 | 'daily life activity'/exp OR 'grooming'/exp OR 'cooking'/exp OR 'drinking'/exp OR 'eating'/exp OR 'personal hygiene'/exp OR 'physical mobility'/exp OR 'functional status'/exp OR 'sitting'/exp OR 'stair climbing'/exp OR 'walking'/exp OR 'locomotion'/exp OR 'motor dysfunction'/exp OR 'physical disability'/exp OR 'incontinence'/exp OR 'toileting'/exp OR 'independent living'/exp OR 'mobilization'/exp OR (adl OR 'activities of daily living*' OR 'daily living activit*' OR 'daily physical activ*' OR 'activity limitation*' OR 'limitation of activit*' OR 'limitation of daily activ*' OR 'independent living*' OR 'everyday function*' OR 'functional abilit*' OR 'functional able*' OR 'functional status' OR 'patient functioning' OR ‘physical abilit*’ OR 'physical able*' OR disability OR 'daily function*' OR 'mobility' OR 'ambulating' OR ambulation OR 'self care' OR selfcare OR eating OR feeding OR drinking OR bathing OR shower* OR wash* OR 'personal care' OR 'personal hygien*' OR cleanliness OR ‘oral hygien*’ OR ‘toothbrushing’ OR 'getting dressed' OR 'getting undressed' OR 'dressing' OR walking OR 'stair climbing' OR 'stairs climbing' OR 'climbing stairs' OR grooming OR toileting OR 'toilet use' OR 'going to the toilet' OR excretion OR 'urine control*' OR 'bowel control*' OR 'controlling bowel*' OR 'bladder control*' OR 'controlling bladder' OR continenc* OR incontinenc* OR 'getting up' OR 'going to bed' OR 'seating' OR transferring):ti,ab,kw | 2,958,987 |
| #2 | 'health data'/exp OR 'medical record'/exp OR 'patient health record information system software'/exp OR ('routinely record*' OR 'routinely collect*' OR 'medical record*' OR 'health record*' OR EHR OR EHRs OR EMR OR EMRs OR 'patient record*' OR 'health data' OR 'routine data' OR 'administrative data' OR 'clinical data' OR 'real world data' OR 'health information exchang*' OR 'medical information exchang*' OR 'health summar*' OR ‘medical histor*’ OR ‘patient histor*’ OR ‘patient coding’):ti,ab,kw | 921,114 |
| #1 | 'natural language processing'/exp OR 'information retrieval'/exp OR 'data mining'/exp OR ('natural language*' OR nlp OR 'text mining' OR textmining OR 'data mining' OR datamining OR ‘information retrieval’ OR ‘information extract*’ OR ‘data extract*’ OR ‘data retriev*’ OR 'machine learning' OR 'deep learning' OR 'active learning' OR 'sentiment analys*' OR 'opinion mining*' OR 'sentiment classification*' OR 'unstructured text*' OR 'unstructured data' OR 'free text*' OR freetext*):ti,ab,kw | 259,324 |

**Cinahl (Ebsco) History and Search Details November 4, 2022**

| **Search** | **Query** | **Results** |
| --- | --- | --- |
| S5 | S4 AND **Limiters** - Published Date: 20120101- | 182 |
| S4 | S1 AND S2 AND S3 | 214 |
| S3 | MH ("Activities of Daily Living+" OR "Grooming+" OR "Meal Preparation+" OR "Oral Hygiene+" OR "Physical Mobility" OR "Functional Status" OR "Sitting" OR "Stair Climbing" OR "Locomotion+" OR "Hygiene" OR "Dental Hygiene" OR "Incontinence+" OR "Eating" OR "Toileting" OR "Walking") OR TI (ADL OR “activities of daily living*” OR “daily living activit*” OR “daily physical activ*” OR “activity limitation*” OR “limitation of activit*” OR “limitation of daily activ*” OR “independent living*” OR “everyday function*” OR ”functional abilit*” OR “functional able*” OR “functional status” OR “patient functioning” OR “physical abilit*” OR “physical able*” OR disability OR “daily function*” OR “mobility” OR “ambulating” OR ambulation OR self-care OR selfcare OR eating OR feeding OR drinking OR bathing OR shower* OR wash* OR “personal care” OR “personal hygien*” OR cleanliness OR “oral hygien*” OR toothbrushing OR “getting dressed” OR “getting undressed” OR “dressing” OR walking OR “stair climbing” OR “stairs climbing” OR “climbing stairs” OR grooming OR toileting OR “toilet use” OR “going to the toilet” OR excretion OR “urine control*” OR “bowel control*” OR “controlling bowel*” OR “bladder control*” OR “controlling bladder” OR continenc* OR incontinenc* OR “getting up” OR “going to bed” OR “seating” OR transferring) OR AB (ADL OR “activities of daily living*” OR “daily living activit*” OR “daily physical activ*” OR “activity limitation*” OR “limitation of activit*” OR “limitation of daily activ*” OR “independent living*” OR “everyday function*” OR ”functional abilit*” OR “functional able*” OR “functional status” OR “patient functioning” OR “physical abilit*” OR “physical able*” OR disability OR “daily function*” OR “mobility” OR “ambulating” OR ambulation OR self-care OR selfcare OR eating OR feeding OR drinking OR bathing OR shower* OR wash* OR “personal care” OR “personal hygien*” OR cleanliness OR “oral hygien*” OR toothbrushing OR “getting dressed” OR “getting undressed” OR “dressing” OR walking OR “stair climbing” OR “stairs climbing” OR “climbing stairs” OR grooming OR toileting OR “toilet use” OR “going to the toilet” OR excretion OR “urine control*” OR “bowel control*” OR “controlling bowel*” OR “bladder control*” OR “controlling bladder” OR continenc* OR incontinenc* OR “getting up” OR “going to bed” OR “seating” OR transferring) | 529,748 |
| S2 | MH ("Routinely Collected Health Data" OR "Medical Records+" OR "Patient Record Systems") OR TI (“routinely record*” OR “routinely collect*” OR “medical record*” OR “health record*” OR EHR OR EHRs OR EMR OR EMRs OR “patient record*” OR “health data” OR “routine data” OR “administrative data” OR “clinical data” OR “real world data” OR “health information exchang*” OR “medical information exchang*” OR “health summar*” OR "medical histor*" OR "patient histor*" OR "patient coding*") OR AB (“routinely record*” OR “routinely collect*” OR “medical record*” OR “health record*” OR EHR OR EHRs OR EMR OR EMRs OR “patient record*” OR “health data” OR “routine data” OR “administrative data” OR “clinical data” OR “real world data” OR “health information exchang*” OR “medical information exchang*” OR “health summar*” OR "medical histor*" OR "patient histor*" OR "patient coding*") | 213,057 |
| S1 | MH ("Natural Language Processing" OR "Data Mining+" OR "Information Retrieval+") OR TI (“natural language*” OR NLP OR “text mining” OR textmining OR “data mining” OR datamining OR “information retriev*” OR “information extract*” OR “data extract*” OR “data retriev*” OR “machine learning” OR “deep learning” OR “active learning” OR “sentiment analys*” OR “opinion mining*” OR “sentiment classification*” OR “unstructured text*” OR “unstructured data” OR free-text* OR freetext*) OR AB (“natural language*” OR NLP OR “text mining” OR textmining OR “data mining” OR datamining OR “information retriev*” OR “information extract*” OR “data extract*” OR “data retriev*” OR “machine learning” OR “deep learning” OR “active learning” OR “sentiment analys*” OR “opinion mining*” OR “sentiment classification*” OR “unstructured text*” OR “unstructured data” OR free-text* OR freetext*) | 50,721 |

**Web of Science Core Collection History and Search Details November 4, 2022**

| **Search** | **Query** | **Results** |
| --- | --- | --- |
| #5 | #4 AND Publication Date: 2012-01-01 to 2023-12-31 | 514 |
| #4 | #1 AND #2 AND #3 | 560 |
| #3 | TS= (“ADL” OR “activities of daily living*” OR “daily living activit*” OR “daily physical activ*” OR “activity limitation*” OR “limitation of activit*” OR “limitation of daily activ*” OR “independent living*” OR “everyday function*” OR ”functional abilit*” OR “functional able*” OR “functional status” OR “patient functioning” OR “physical abilit*” OR “physical able*” OR “disability” OR “daily function*” OR “mobility” OR “ambulating” OR ambulation OR self-care OR selfcare OR “eating” OR “feeding” OR “drinking” OR “bathing” OR shower* OR wash* OR “personal care” OR “personal hygien*” OR cleanliness OR “oral hygien*” OR toothbrushing OR “getting dressed” OR “getting undressed” OR “dressing” OR walking OR “stair climbing” OR “stairs climbing” OR “climbing stairs” OR “grooming” OR “toileting” OR “toilet use” OR “going to the toilet” OR excretion OR “urine control*” OR “bowel control*” OR “controlling bowel*” OR “bladder control*” OR “controlling bladder” OR continenc* OR incontinenc* OR “getting up” OR “going to bed” OR “seating” OR “transferring”) | 2,175,370 |
| #2 | TS= (“routinely record*” OR “routinely collect*” OR “medical record*” OR “health record*” OR “HER” OR “EHRs” OR “EMR” OR “EMRs” OR “patient record*” OR “health data” OR “routine data” OR “administrative data” OR “clinical data” OR “real world data” OR “health information exchang*” OR “medical information exchang*” OR “health summar*” OR "medical histor*" OR "patient histor*" OR "patient coding*") | 601,809 |
| #1 | TS = (“natural language*” OR “NLP” OR “text mining” OR textmining OR “data mining” OR datamining OR “information retriev*” OR “information extract*” OR “data extract*” OR “data retriev*” OR “machine learning” OR “deep learning” OR “active learning” OR “sentiment analys*” OR “opinion mining*” OR “sentiment classification*” OR “unstructured text*” OR “unstructured data” OR free-text* OR freetext*) | 423,822 |

**Scopus History and Search Details November 4, 2022**

| **Search** | **Query** | **Results** |
| --- | --- | --- |
| #5 | #4 AND PUBYEAR AFT 2011 | 736 |
| #4 | #1 AND #2 AND #3 | 898 |
| #3 | TITLE-ABS (“ADL” OR “activities of daily living*” OR “daily living activit*” OR “daily physical activ*” OR “activity limitation*” OR “limitation of activit*” OR “limitation of daily activ*” OR “independent living*” OR “everyday function*” OR ”functional abilit*” OR “functional able*” OR “functional status” OR “patient functioning” OR “physical abilit*” OR “physical able*” OR “disability” OR “daily function*” OR “mobility” OR “ambulating” OR ambulation OR self-care OR selfcare OR “eating” OR “feeding” OR “drinking” OR “bathing” OR shower* OR wash* OR “personal care” OR “personal hygien*” OR cleanliness OR “oral hygien*” OR toothbrushing OR “getting dressed” OR “getting undressed” OR “dressing” OR walking OR “stair climbing” OR “stairs climbing” OR “climbing stairs” OR “grooming” OR “toileting” OR “toilet use” OR “going to the toilet” OR excretion OR “urine control*” OR “bowel control*” OR “controlling bowel*” OR “bladder control*” OR “controlling bladder” OR continenc* OR incontinenc* OR “getting up” OR “going to bed” OR “seating” OR “transferring”) OR AUTHKEY (“ADL” OR “activities of daily living*” OR “daily living activit*” OR “daily physical activ*” OR “activity limitation*” OR “limitation of activit*” OR “limitation of daily activ*” OR “independent living*” OR “everyday function*” OR ”functional abilit*” OR “functional able*” OR “functional status” OR “patient functioning” OR “physical abilit*” OR “physical able*” OR “disability” OR “daily function*” OR “mobility” OR “ambulating” OR ambulation OR self-care OR selfcare OR “eating” OR “feeding” OR “drinking” OR “bathing” OR shower* OR wash* OR “personal care” OR “personal hygien*” OR cleanliness OR “oral hygien*” OR toothbrushing OR “getting dressed” OR “getting undressed” OR “dressing” OR walking OR “stair climbing” OR “stairs climbing” OR “climbing stairs” OR “grooming” OR “toileting” OR “toilet use” OR “going to the toilet” OR excretion OR “urine control*” OR “bowel control*” OR “controlling bowel*” OR “bladder control*” OR “controlling bladder” OR continenc* OR incontinenc* OR “getting up” OR “going to bed” OR “seating” OR “transferring”) | 2,766,055 |
| #2 | TITLE-ABS (“routinely record*” OR “routinely collect*” OR “medical record*” OR “health record*” OR “HER” OR “EHRs” OR “EMR” OR “EMRs” OR “patient record*” OR “health data” OR “routine data” OR “administrative data” OR “clinical data” OR “real world data” OR “health information exchang*” OR “medical information exchang*” OR “health summar*” OR "medical histor*" OR "patient histor*" OR "patient coding*") OR AUTHKEY (“routinely record*” OR “routinely collect*” OR “medical record*” OR “health record*” OR “HER” OR “EHRs” OR “EMR” OR “EMRs” OR “patient record*” OR “health data” OR “routine data” OR “administrative data” OR “clinical data” OR “real world data” OR “health information exchang*” OR “medical information exchang*” OR “health summar*” OR "medical histor*" OR "patient histor*" OR "patient coding*") | 910,020 |
| #1 | TITLE-ABS (“natural language*” OR “NLP” OR “text mining” OR textmining OR “data mining” OR datamining OR “information retriev*” OR “information extract*” OR “data extract*” OR “data retriev*” OR “machine learning” OR “deep learning” OR “active learning” OR “sentiment analys*” OR “opinion mining*” OR “sentiment classification*” OR “unstructured text*” OR “unstructured data” OR free-text* OR freetext*) OR AUTHKEY (“natural language*” OR “NLP” OR “text mining” OR textmining OR “data mining” OR datamining OR “information retriev*” OR “information extract*” OR “data extract*” OR “data retriev*” OR “machine learning” OR “deep learning” OR “active learning” OR “sentiment analys*” OR “opinion mining*” OR “sentiment classification*” OR “unstructured text*” OR “unstructured data” OR free-text* OR freetext*) | 863,334 |
